# Neural peer pressure: intercellular dynamics and emergent phenotypes in the mosaic Rett syndrome brain

**DOI:** 10.1186/s12964-026-02987-w

**Published:** 2026-06-16

**Authors:** Jarno Koetsier, Chris P. Reutelingsperger, Leopold M.G. Curfs, Lars M.T. Eijssen, Nasim Bahram Sangani

**Affiliations:** 1 Department of Biochemistry, Cardiovascular Research Institute Maastricht (CARIM), Maastricht University, Maastricht, The Netherlands; 2 GKC, Maastricht University Medical Centre, Maastricht, The Netherlands; 3 Department of Psychiatry and Neuropsychology, School for Mental Health and Neuroscience (MHeNs), Maastricht University, Maastricht, The Netherlands; 4 Department of Translational Genomics, Maastricht University, Maastricht, The Netherlands

**Keywords:** Rett syndrome, Mosaicism, Intercellular communication, Non-cell-autonomous effects, Emergent behavior

## Abstract

During female embryogenesis, each cell randomly inactivates either the maternal or paternal X chromosome, creating a natural mix, or mosaic, of cells with a different active X chromosome. In X-linked neurological disorders such as Rett syndrome, this results in a brain composed of two cell populations: one expressing the mutant allele and one expressing the wildtype allele. Rather than behaving as independent entities, these populations influence one another through the exchange of extracellular vesicles, free-floating biomolecules, and direct cell–cell contacts. The bidirectional communication between mutant and wildtype cells can give rise to emergent behaviors that cannot be explained by cell-autonomous mechanisms alone. Particularly, rather than a simple dichotomy of two discrete cell states, emerging evidence suggests that the mosaic Rett syndrome brain contains a spectrum of cells with intermediate phenotypes. Hence, shifting focus from isolated cellular effects to cell population-level dynamics will be crucial for advancing our understanding of mosaic conditions and developing effective therapeutic strategies.

## Introduction

Every female mammal is a natural mosaic. Early in female embryonic development, one of the two X chromosomes in each cell is randomly silenced through X-chromosome inactivation (XCI), a process that equalizes X-linked gene dosage between the sexes [1]. In humans, XCI is a gradual process that is thought to start at around the 8-cell stage with the expression of XIST, a long non-coding RNA (lncRNA) that serves as the key initiator of XCI [2–4]. At this stage, an XIST cloud forms around both X chromosomes, leading to the recruitment of repressive histone modifications [5, 6]. As development progresses, asymmetry emerges: the future active X chromosome represses XIST expression and erases the repressive marks, whereas the future inactive X chromosome maintains XIST coating and the associated repressive epigenetic modifications, ultimately condensing into the Barr body [7, 8]. It takes until after implantation for the XCI status to be completely established [1, 6, 7, 9]. For a more comprehensive overview of XCI mechanisms, see reviews by Loda et al. [1] and Alfeghaly & Rougeulle [10].

The outcome of XCI is a cellular mosaic in which each cell expresses either the maternal or the paternal X chromosome. In adult tissues, this mosaic typically exhibits a relatively homogeneous spatial distribution, rather than the “patchy” pattern observed in the placenta, possibly as a result of cell migration during development [11]. In females carrying pathogenic X-linked variants, XCI mosaicism leads to a coexistence of mutant and wildtype cell populations, resulting in different cellular phenotypes despite a shared genotype. Classic examples include Duchenne muscular dystrophy (DMD), hemophilia A and B, fragile X syndrome (FXS), and Rett syndrome (RTT). Among these X-linked disorders, RTT—a rare neurodevelopmental disorder caused by loss-of-function mutations in the *MECP2* gene [12]—stands out because it almost exclusively affects females. Hemizygous RTT-causing *MECP2* mutations in males typically cause pre- or perinatal lethality [13], so surviving patients are mostly heterozygous females. In these females, XCI generates a brain-wide mosaic of wildtype and *MECP2*-mutant expressing neurons and glia. Hence, the RTT brain provides a natural system to study how (epi)genetically distinct cell populations influence one another’s function. Moreover, understanding mosaicism is critical for explaining both the variability of clinical symptoms and the cellular mechanisms of RTT.

**Fig. 1:**
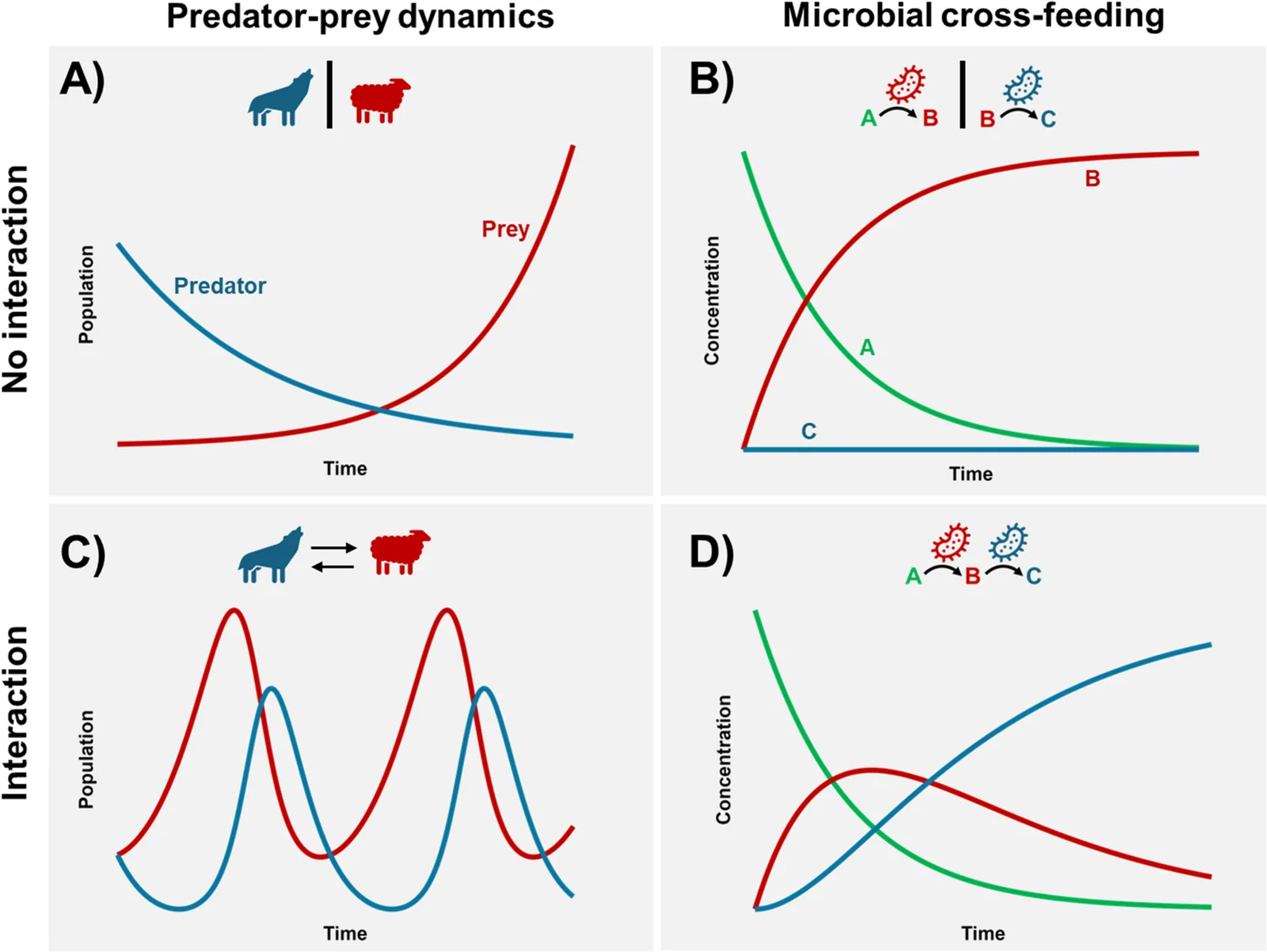
Two interacting populations can give rise to emergent behavior, such as predator-prey oscillations (**C**) and microbial cross-feeding (**D**). These emergent behaviors cannot be explained by studying each population in isolation (**A** and **B**)

The interplay between the two cell populations in the RTT brain can generate emergent properties—phenotypes that cannot be predicted by examining each cell population individually. Emergent behavior of two interacting populations can be seen across biological systems, from predator-prey oscillations [14] to mutualistic cross-feeding in microbes [15] (Fig. 1). These examples teach us that studying each individual population is not sufficient to understand the collective behavior. Hence, this review shifts the focus away from the cell-autonomous effects of MeCP2 loss-of-function and instead focuses on the interactions between mutant and wildtype cells. Specifically, we aim to explore how these two populations communicate, influence one another’s phenotypes, and ultimately shape the functional landscape of the mosaic RTT brain.

## Rett syndrome is a heterogeneous disease

Before exploring how mutant and wildtype cells interact within the mosaic RTT brain, it is important to first outline the clinical features of the disease. After an apparently normal period of early development, typically lasting 6 to 18 months, patients with RTT experience a phase of developmental stagnation [16, 17]. This phase is associated with microcephaly, growth retardation, weight loss, and hypotonia. During the second year of life, this stage is followed by a phase of rapid regression during which acquired motor and communication skills are lost. As the disease progresses, most patients with RTT also develop multisystem complications, including cardiac, gastrointestinal, and respiratory anomalies, as well as life-threatening seizures.

More than 90% of patients with RTT carry a pathogenic variant in the X-linked *MECP2* gene [18, 19], a multifunctional regulator of chromatin structure, transcription, RNA splicing, and DNA damage repair [20]. Despite its monogenic origin, RTT exhibits remarkable clinical heterogeneity, with severity and spectrum of symptoms varying widely among individuals [21]. For instance, not all patients will develop seizures or lose the ability to walk or communicate. Moreover, some females harboring a pathogenic *MECP2* mutation even remain asymptomatic [22]. This heterogeneity arises from two sources: (1) genotype, encompassing both *MECP2* mutation type and genetic background, and (2) XCI skewness, including the degree and spatial distribution of XCI in the brain.

Both the *MECP2* mutation type and genetic background contribute substantially to interindividual variability in disease severity. Generally, large deletions or early-truncating *MECP2* mutations cause more severe phenotypes than missense or late-truncating mutations that partially preserve MeCP2 protein function [23, 24]. For instance, in contrast to patients with RTT carrying early-truncating mutations, those with the missense R306C mutation typically begin regressing only after 30 months of age and do not need assistance with ambulation [23]. Beyond *MECP2* itself, the patient’s genetic background can further modulate disease severity. Particularly, genetic variations in several genes, such as *BDNF* [25], *MTHFR* [26], and *CROCC* [27], have been suggested as potential modifiers of RTT outcomes. Renieri et al. even went as far as to propose a digenic model for RTT, where a pathogenic variant in a second gene determines whether a *MECP2* mutation remains silent or leads to RTT [28].

**Fig. 2:**
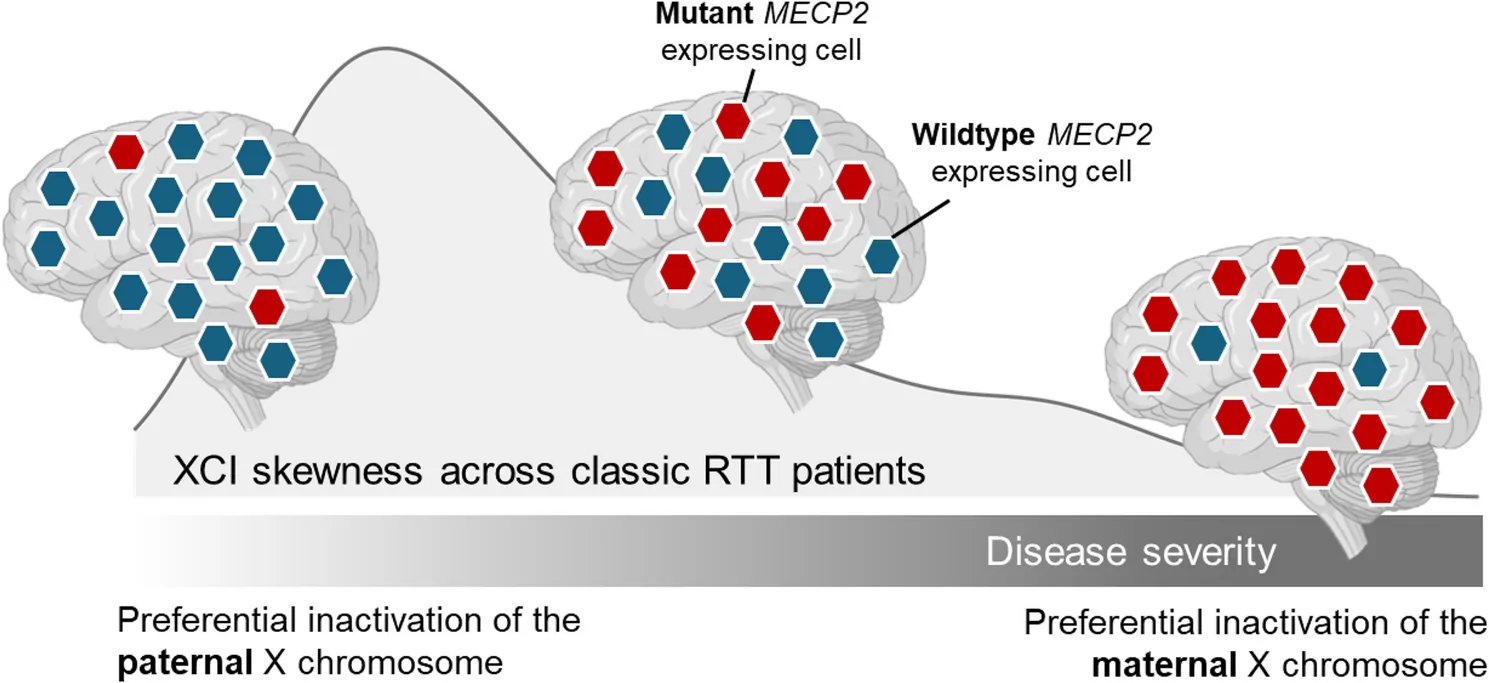
Schematic representation of XCI skewing in RTT. Because RTT-causing *MECP2* mutations are commonly of paternal origin [29, 30], preferential inactivation of the maternal X chromosome is associated with increased disease severity. The density plot depicts the distribution of XCI skewness among patients with classic RTT from the *RTT and RTT-Related Disorders Natural History Study (RNHS)* [32], with probability density shown on the y-axis. Notably, most patients with RTT exhibit preferential inactivation of the paternal X chromosome, reflecting a relatively favorable XCI pattern

A final major source of symptom variability in RTT relates to XCI skewness, a feature that distinguishes X-linked disorders like RTT from other monogenic diseases. In approximately 95% of the sporadic RTT cases, the *de novo MECP2* mutation is of paternal origin [29, 30]. Hence, skewing toward the wildtype maternal X chromosome typically results in milder RTT phenotypes, whereas skewing toward the mutant paternal X chromosome exacerbates symptoms [31, 32] (Fig. 2). However, as discussed later in this article, the relationship between XCI skewness and disease severity seems to be non-linear and dependent on the spatial distribution across brain regions, highlighting the complex mosaic nature of RTT pathology.

## Non-cell-autonomous effects of mutant cells

**Fig. 3:**
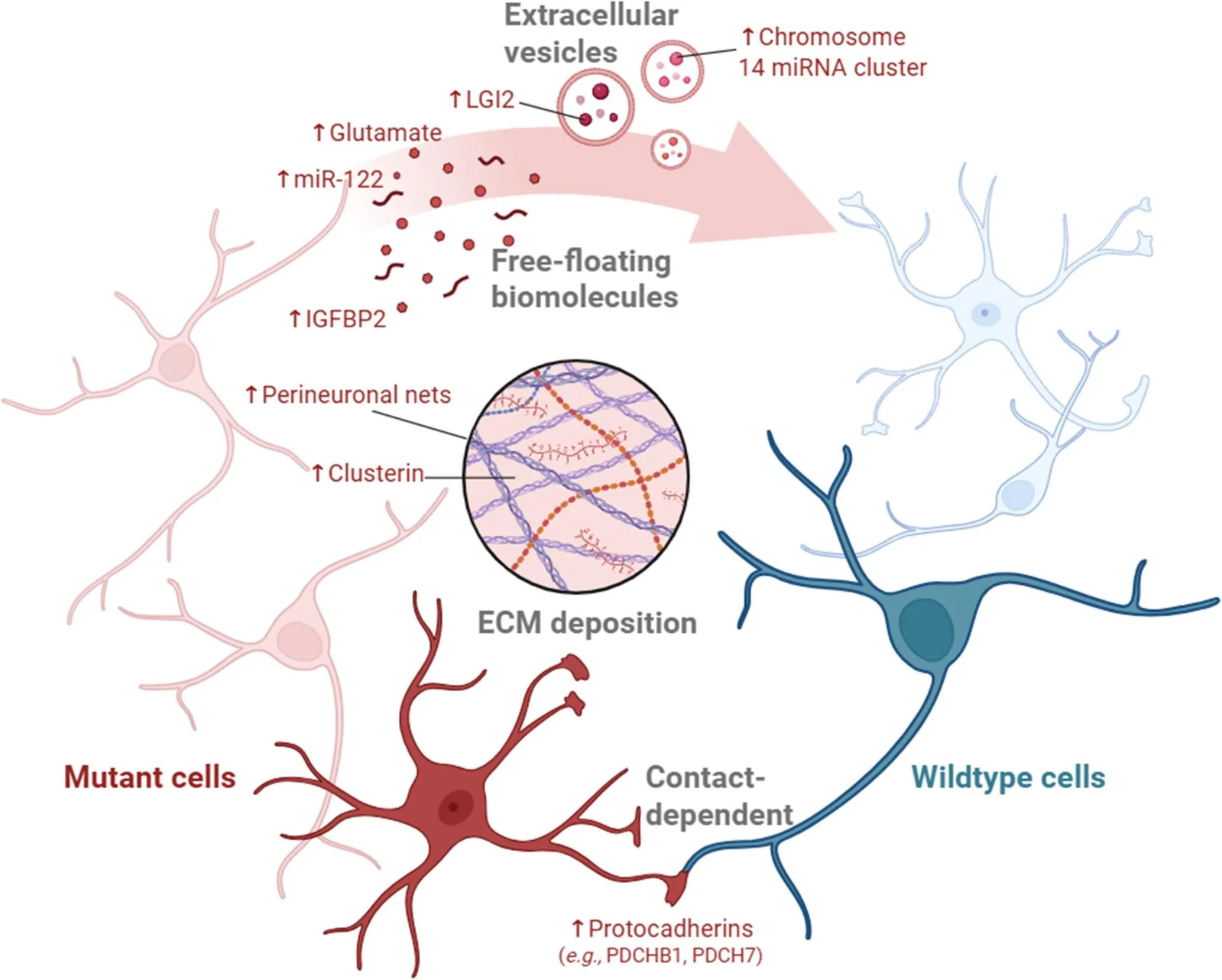
Schematic overview of “disease” signals produced by mutant *MECP2*-expressing neural cells. These “disease” signals include EV-derived and free-floating proteins, miRNAs, and other biomolecules, as well as extracellular matrix components and contact-dependent signals. *In vitro and in vivo* studies demonstrated that these “disease” signals can negatively affect morphology and function neighboring wildtype cells within a mosaic cellular environment. Image created with BioRender.com

In a mosaic environment, mutant cells can communicate with nearby wildtype cells through both paracrine and contact-dependent signaling pathways (Fig. 3). Among the paracrine routes, extracellular vesicle (EV)-mediated signaling has been well studied in neurological conditions. EVs are particles composed of a lipid bilayer enclosing biomolecules such as RNAs, proteins, and metabolites. They are released into the extracellular environment either by budding from the plasma membrane or via fusion of the outer membrane of multivesicular bodies with the plasma membrane [33, 34]. After release, EVs can move through the extracellular space to reach their target cells. At the target cells, EVs can fuse with the plasma membrane, induce an intracellular signaling cascade through ligand-receptor interactions, or be internalized through endocytosis [35, 36].

Previous studies have demonstrated that EV-mediated communication is altered in RTT. Specifically, the protein content of both plasma-derived EVs from patients with classic RTT [37] and EVs from hemizygous *MECP2*-mutant (p.Q83X) human induced pluripotent stem cell (hiPSC)-derived neural cell cultures [38] was found to be altered. In the *MECP2*-mutant neural cell cultures, proteins that are involved in neural development, such as LGI2, showed increased levels in EVs [38]. Interestingly, not all alterations were a direct reflection of changes at the cellular level, suggesting the presence of differential EV packaging mechanisms in mutant cells. Besides the protein content, the miRNA content of EVs was also found to be altered in female *MECP2*-mutant (p.R255X) hiPSC-derived brain organoids [39]. The EV-derived miRNAs with a changed abundance include members of the hsa-miR-302/367 family and the chromosome 14 miRNA cluster, which are both important regulators of neural development. Interestingly, some of the changes in these EV-derived miRNAs were reported to be dependent on the developmental time point and brain region, demonstrating that signaling by mutant cells is highly dynamic and changes throughout neurodevelopment. Finally, a cross-tissue transcriptome-wide meta-analysis showed that *ITGB4*, a gene essential for exosome secretion by astrocytes, exhibits increased expression in RTT [40].

Mutant cells further influence the phenotype of surrounding cells through non-vesicle-mediated paracrine signaling. Astrocytes in particular have been well-studied in this context. Two studies observed alterations in the secretome – the collection of secreted proteins – of astrocytes from hemizygous *Mecp2*-null (Mecp2^−/y^) mice [41, 42]. Both found the upregulation of extracellular matrix-related proteins, such as the extracellular chaperone Clusterin (Clu). These observations may indicate that *Mecp2*-null cells are actively remodeling the extracellular matrix. This is in line with findings from Carstens et al., who observed an increased deposition of perineuronal nets – specialized extracellular matrix structures – in the hippocampal area CA2 of a patient with RTT and *Mecp2*^−/y^ mice [43]. The increase in perineuronal net formation may impact wildtype neurons by inhibiting synaptic plasticity [44].

In addition to extracellular matrix-related proteins, the study by Caldwell et al. found an increase in the release of IGFBP2 across multiple neurodevelopmental disorders, including RTT, FXS, and Down syndrome [41]. IGFBP2 is an inhibitor of IGF and can thereby prevent normal neurite outgrowth in these neurodevelopmental disorders. Besides proteins, plasma levels of free-floating brain-enriched miRNAs were found to be altered in *Mecp2*^−/y^ and heterozygous *Mecp2*-null (*Mecp2*^­−/+^) mice as well as in human patients with RTT [45]. Interestingly, among these miRNAs were miR-132 and miR-122, well-described miRNAs that have essential roles in neural development and have been associated with multiple neuropathologies [46, 47]. Finally, increased glutamate release by *Mecp2*^−/y^ microglia has been shown to have dendritotoxic and synaptotoxic effects on wildtype neurons [48]. Similarly, the ability of homozygous *Mecp2*-null (*Mecp2*^−/−^) microglia to induce synaptic deficits has also been reported by Tiberi et al. [49]. Nevertheless, microglial *Mecp2* expression alone was not sufficient to improve the phenotype of *Mecp2*^−/y^ mice [50].

Besides paracrine signaling, contact-mediated communication between the cells in the central nervous system is essential for the normal development and function of neurons [51], astrocytes [52], and microglia [53]. Research by Maezawa et al. showed that the non-cell-autonomous effects of *Mecp2*^−/+^ astrocytes are partly mediated by gap junctions, as their inhibition was able to reduce the spread of MeCP2 deficiency in cell culture [54]. Furthermore, an in vitro study by Miyake et al. demonstrated that MeCP2 directly regulates the expression of protocadherin genes *PDCHB1* and *PDCH7*, leading to an upregulated expression of these cell-cell adhesion molecules in *MECP2*-knockdown SH-SY5Y cells and the frontal cortex of *Mecp2*^−/y^ mice [55]. Further evidence of aberrant contact-dependent communication in RTT is provided by Yoo et al., who found a downregulation of L1CAM, a cell-cell adhesion protein, in hemizygous *MECP2*-mutant (p.Q83X) hiPSC-derived neural progenitor cells (NPCs) [56]. Together, these findings suggest that mutant cells may impact the phenotype of wildtype cells through aberrant contact-dependent interactions.

Collectively, studies indicate that mutant cells have altered paracrine and contact-dependent signaling properties through which they can impact the phenotype of neighboring wildtype cells (Fig. 3). The non-cell-autonomous impact on the phenotype of wildtype cells has been experimentally shown by several studies. In vitro studies found that human stem cell-derived *MECP2*-mutant and mouse *Mecp2*^−/y^ astrocytes (and their conditioned medium) negatively impact the growth, morphology, and electrophysiological properties of wildtype neurons [57–59]. Similarly, the absence of MeCP2 in mouse astrocytes prevents normal neuronal synaptic signaling in response to astrocyte stimulation [60]. Furthermore, when co-cultured with hemizygous *MECP2*-mutant human embryonic stem cell (hESC)-derived neurons, wildtype astrocytes exhibit reduced structural complexity, demonstrating a bidirectional impact of astrocytes and neurons [59].

 In vivo observations echo these findings. In mosaic RTT mouse model brains (*Mecp2*^−/+^), wildtype neurons have a reduced dendritic complexity and spine density as well as smaller soma and nuclear size than in a purely wildtype environment [61–63]. Electrophysiological properties of wildtype neurons, such as inhibitory postsynaptic current frequency, were also found to be different in mosaic (*Mecp2*^−/+^) versus non-mosaic mouse brains (*Mecp2*^+/+^) [64]. Finally, the transcriptome of wildtype neurons in a mosaic mouse brain (*Mecp2*^−/+^) was shown to be remarkably different from neurons in a purely wildtype environment [65].

## Can healthy signals rescue mutant cells?

Given that mutant cells can adversely affect the phenotype of wildtype cells, it is natural to ask whether the reverse might also occur: can wildtype cells positively influence the phenotype of co-existing mutant cells? Williams et al. demonstrated that, compared to *MECP2*-mutant astrocytes, wildtype astrocytes have a positive impact on the morphology of co-cultured mutant interneurons [57]. Similarly, the re-expression of *Mecp2* astrocytes in *Mecp2*^−/y^ and *Mecp2*^−/+^ mice was shown to significantly improve disease phenotype by restoring the morphology of neurons and the neuronal expression of VGLUT, a protein involved in excitatory neurotransmission [66]. Although these studies found that, compared to mutant astrocytes, wildtype astrocytes have a positive effect on mutant neurons, it is unclear whether this positive effect is because of the active production of “positive” signals by wildtype astrocytes or because of the absence of “negative” signals produced by mutant cells.

The study by Sharma et al. provided us with evidence showing that wildtype cells actively produce “positive” signals [38]. Particularly, the authors demonstrated that, compared to no treatment, treating hemizygous *MECP2*-mutant (p.Q83X) neural cell cultures with exosomes from healthy neuronal cultures can restore neuronal development and function. This effect might be mediated by exosomal proteins, as wildtype exosomes contain proteins involved in neural development, such as ATRN and LGALS1, which have reduced levels in exosomes from *MECP2*-mutant cell culture [38]. Another protein that may mediate the beneficial effects of wildtype cells is BDNF, a growth factor produced and released by neurons, microglia, and astrocytes [67]. BDNF levels have been reported to be higher in wildtype compared to *Mecp2*^−/y^ and *Mecp2*^−/+^ mouse models [68]. Increasing endo- and exogenous BDNF levels can (partially) rescue morphological and functional deficits of *Mecp2-*defient neurons (e.g.,* Mecp2*-knockdown [69] and *Mecp2*^*−/y*^ [70] neurons). Hence, the production of BDNF might be another way by which wildtype neurons, microglia, and astrocytes improve the phenotype of surrounding mutant cells.

**Fig. 4:**
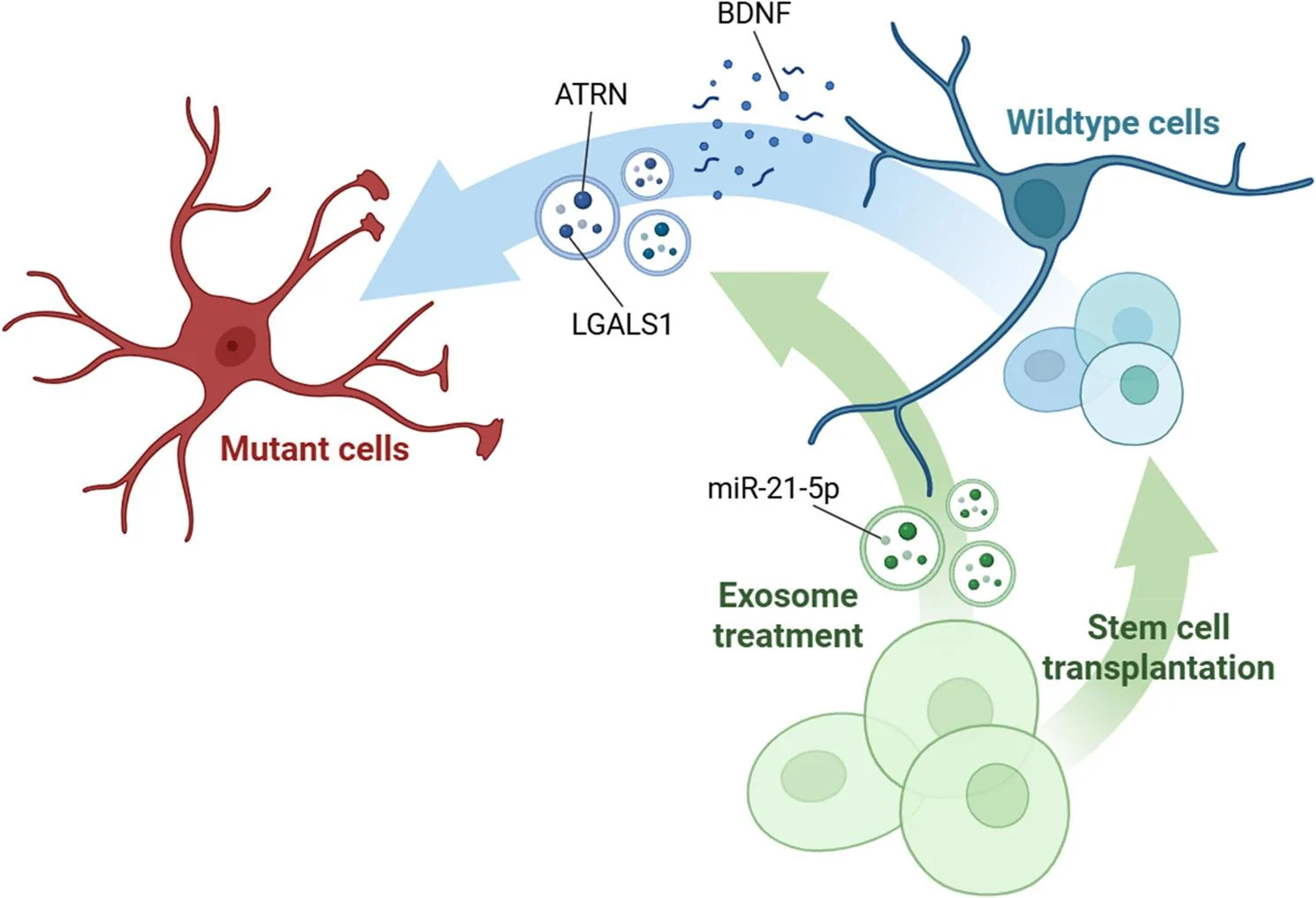
Schematic overview of “healthy” signals produced by wildtype *MECP2*-expressing neural cells and their potential therapeutic use. In vitro and in vivo studies have shown that wildtype neural cells can produce and excrete molecules that partially restore function and morphology of mutant *MECP2*-expressing cells. These candidate “healthy” signals include, among others, free-floating (e.g., BDNF) and EV-derived (e.g., ATRN and LGALS1) proteins. Experimental preclinical studies demonstrated that stem cell transplantation and exosome treatment make use of “healthy” signaling to promote neurogenesis and improve RTT-like symptoms in mice. These findings highlight a promising, yet still preclinical, therapeutic strategy. Image created with BioRender.com

The positive impact of factors secreted by wildtype cells can be leveraged for developing treatment approaches (Fig. 4). An example of this is the transplantation of cells that are able to secrete factors that support neural development and function. Mesenchymal stem cells, for instance, can secrete, among others, extracellular vesicles, cytokines, chemokines, extracellular matrix components, and growth factors that have anti-inflammatory, pro-angiogenic, and anti-apoptotic effects [71, 72]. Because of these beneficial effects, stem cell transplantation has been suggested as a promising therapeutic avenue for autism spectrum disorders [71]. Such a therapeutic strategy might be beneficial for RTT as well. For instance, the transplantation of BDNF-secreting mesenchymal stem cells in 6-week-old *Mecp2*^−/y^ mice was shown to improve neurogenesis and synaptogenesis [73]. Similarly, NPC transplantation in symptomatic 6-to-7-week-old *Mecp2*^−/y^ mice was found to extend lifespan and improve RTT-like symptoms [74]. In vitro experiments of this study revealed that NPCs secrete factors that increase BDNF levels and synaptic density of *in vitro Mecp2*-knockout neurons. Notably, both wildtype and *Mecp2*-knockout NPCs could achieve this improvement in synaptic density, suggesting that NPCs do not require MeCP2 to exert their beneficial effects. However, it was demonstrated that this therapeutic effect is dependent on the environment of the NPCs; only NPCs co-cultured with knockout neurons will produce factors that can restore synaptic density.

As an alternative to stem cell transplantation, Pan et al. showed that cerebral injection with exosomes from urine-derived stem cells for three weeks promotes neurogenesis and alleviates RTT-like symptoms of hemizygous *Mecp2*-mutant (*Mecp2*^tm1Hzo/y^) mice [75]. Exosomal miR-21-5p was found to be responsible for this therapeutic effect. In vitro experiments highlighted that exosomal miR-21-5p targets Epha4 and promotes differentiation of RTT neural stem cells. Nevertheless, the short half-life and absence of standardized protocols for exosome collection currently limit the clinical applicability of exosome-based treatment strategies [76].

## Mosaicism is more than a two-cell state

**Fig. 5:**
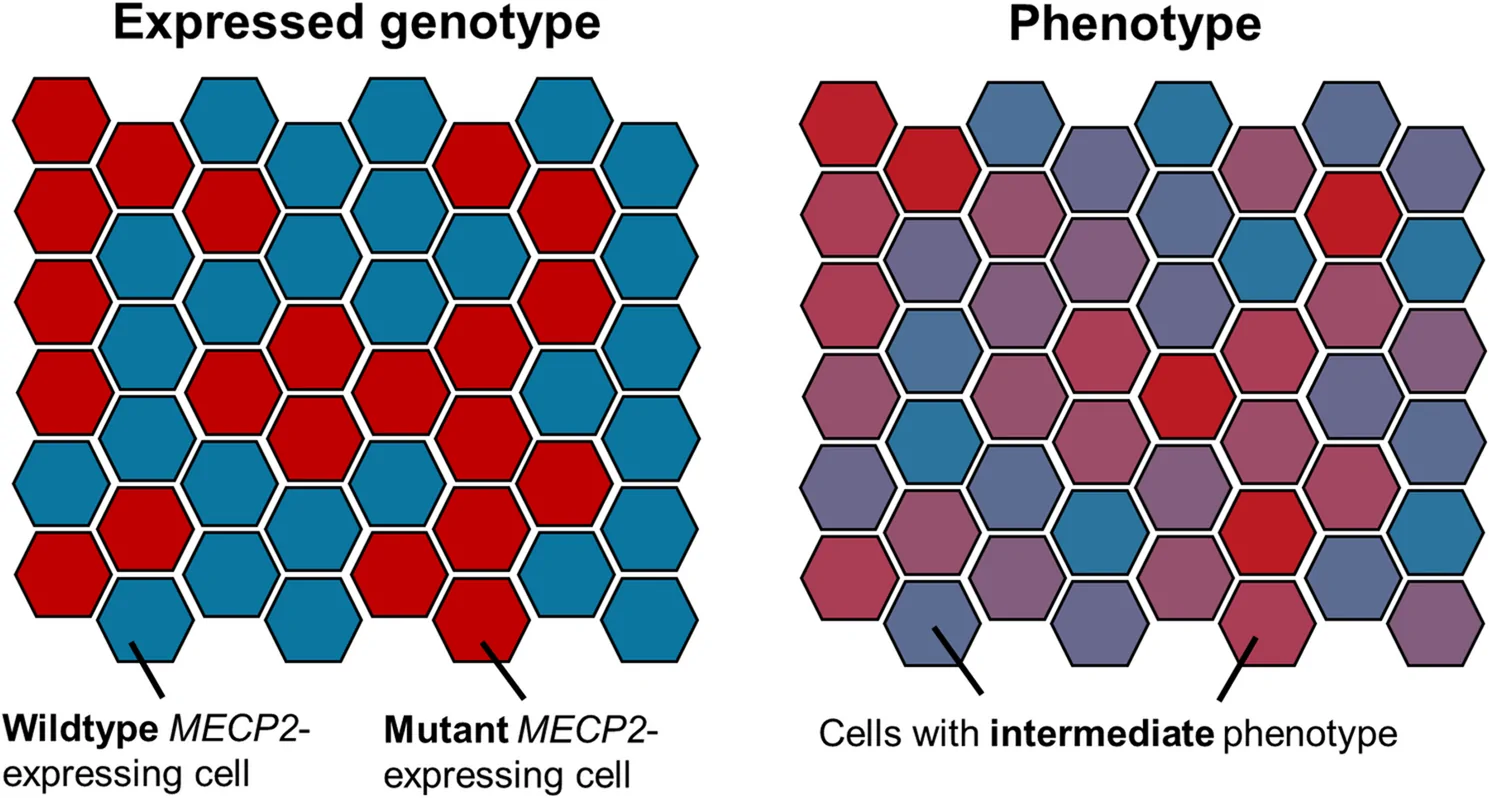
Proposed phenotypic convergence in mosaic RTT tissue. Emerging evidence suggests that in a mosaic environment, intercellular communication may drive wildtype and *MECP2*-mutant expressing cells to adopt intermediate phenotypes, both shifting away from their original mutant or wildtype states (i.e., phenotypic convergence)

Due to intercellular communication, the phenotype of a cell in a mosaic tissue is an intermediate phenotype that determined not only by its expressed genotype (i.e., expression of the mutant or wildtype *MECP2* allele) but also by molecules from its local environment, such as EVs and proteins produced by neighboring cells. A striking example of this comes from a study by Sharifi et al., who observed that the transcriptomic profile of wildtype cells in the mosaic brain was more similar to the mutant cells in the same brain than to wildtype cells in a non-mosaic context [65]. This finding highlights that the cellular environment can have a greater influence on cellular phenotype than the cell’s expressed genotype. The mutant-like phenotype of wildtype cells in a mosaic environment was further highlighted by Braunschweig et al., who showed that MeCP2 expression in wildtype cells inversely correlated with the percentage of MeCP2-negative cells [77]. So, rather than a simple dichotomy of two discrete cell states (wildtype vs. mutant), the mosaic RTT brain contains a spectrum of cells with intermediate phenotypes (Fig. 5). The specific phenotype of any given cell is influenced by its expressed genotype and the local degree of XCI skewness, causing it to lean more toward a “wildtype” or “mutant” state.

Although adult tissues often display mosaic XCI distribution, not a “patchy” one like the placenta [11], significant variation in the local degree of XCI skewness still exists [78, 79] and is relevant to the disease phenotype. Wither et al. showed in heterozygous RTT mouse models that regional differences in MeCP2 expression levels correlate with disease phenotype [80]. For instance, cortical MeCP2 expression negatively correlated with gross symptom severity, while hippocampal MeCP2 levels positively correlated with activity behavior. Interestingly, associations between regional XCI skewness and behavioral phenotype have also been observed in FXS mouse models [78]. Interindividual differences in the spatial distribution of XCI skewness may explain the variability in clinical phenotypes between monozygotic RTT twins with a similar degree of XCI skewness in blood [81].

In addition to spatial differences in XCI skewness, temporal differences in XCI skewness may also occur. Changes in XCI skewness over time are referred to as secondary skewing [82]. Secondary skewing occurs when one cell population has a higher rate of proliferation and/or a lower rate of cell death. Although cellular crosstalk reduces the phenotypic differences between wildtype and mutant cell populations, previous research suggests that there might still be a selective advantage of wildtype cells over mutant cells in a mosaic environment. Particularly, in vivo and in vitro mouse studies observed a decrease in the mutation-to-wildtype ratio over time [83–85]. Such a selective advantage could also explain the favorable XCI skewing observed in most patients with RTT [86, 87] (Fig. 2).

Together, rather than a simple two-cell state, the mosaic RTT brain contains a range of intermediate phenotypes that change over space and time. As the proportion of wildtype cells increases, not only does the overall fraction of healthy cells rise, but both populations adopt a comparatively healthier phenotype due to a shift in the balance of non-cell autonomous signals. This cooperative effect may lead to a non-linear relationship between XCI skewing and clinical severity in RTT. Supporting this idea, Carrette et al. demonstrated that even a modest 5–10% increase in wildtype MeCP2 protein levels in the brain extended the lifespan of heterozygous *Mecp2*-null mice by five- to eightfold [88].

Although most evidence suggests that wildtype cells adopt a more mutant-like phenotype under mosaic conditions, this may not hold for all cellular characteristics and across all mosaic ratios. In particular, many biological stressors follow a biphasic dose-response relationship, whereby low doses can stimulate or enhance physiological functions, whereas high doses disrupt homeostasis and induce harmful effects [89]. This phenomenon is known as hormesis and may also operate in mosaic environments. For instance, glutamate, which is secreted in elevated levels by *Mecp2-null* microglia [48], is known to have such hormetic properties [90]. Under this framework, a low proportion of mutant cells could generate non-cell-autonomous signals that paradoxically confer benefits to neighboring wildtype cells. However, it is important to note that, to date, no studies have reported such hormetic effects on the phenotype of wildtype cells and, leaving this as an open question for future research.

## Challenges for disease modeling

So far, we have seen that cellular mosaicism in RTT is not a minor nuance but a central feature of its pathophysiology. Crosstalk among wildtype and *MECP2*-mutant neurons, astrocytes, oligodendrocytes, and microglia produce profound non-cell-autonomous effects, meaning that studying these cell populations individually can obscure emergent phenotypes arising from their interactions. For example, male non-mosaic hemizygous *Mecp2*-mutant mice, unlike female mosaic heterozygous *Mecp2-*mutant mice, fail to recapitulate the gut microbiome alterations and inflammatory profiles observed in most patients with RTT [91].

Despite their limited translational fidelity, non-mosaic hemizygous male RTT models are commonly used both in vitro (e.g., [92]) and in vivo (e.g., [93–96]) settings. Even when female cell lines are employed, mosaic conditions are avoided by separating mutant and wildtype *MECP2*-expressing clones into distinct experimental groups (e.g., [39, 97–99]). The main reason for using such non-mosaic models is that they reflect a simple binary state: mutant versus wildtype. In contrast, mosaic models represent a continuum, shaped by the degree of XCI skewing and the spatial distribution of mutant and wildtype cells. This leads to a relatively large variability in the outcome variable. For instance, RTT-like phenotypes in mosaic female mice, such as body tremor and stereotypic forepaw movements, are strongly associated with XCI ratio [85].

There are also more practical reasons to work with non-mosaic models. For instance, hemizygous male *Mecp2*-mutant/knockout mouse models are often favored because of the earlier and more severe disease onset compared to females [93, 100, 101]. Moreover, in vitro studies often employ male *MECP2*-knockout or -mutant hiPSC and hESC lines due to concerns over XCI erosion. Particularly, during prolonged culture, female pluripotent stem cells may gradually lose XIST RNA coating of the inactive X chromosome, leading to reactivation of silenced genes in the affected regions (e.g., [102–104]). This erosion persists upon differentiation [105, 106], resulting in bi-allelic expression of X-linked genes. If XCI erosion affects the *MECP2* locus, mosaicism is effectively lost because cells express both the mutant and wildtype allele. Moreover, XCI erosion, in general, distorts the overall proteome [107], reducing the physiological validity of the model. To mitigate this, researchers must keep cells at low passage, avoid LiCl in culture media [108], and perform rigorous XCI quality control (e.g., analysis of XIST expression, H3K27me3 foci, DNA methylation, and allele-specific expression) [102, 103].

## Conclusions and perspectives

Over the past decade, significant progress has been made in understanding how mutant and wildtype cells within the RTT brain influence one another. Rather than existing as independent populations, these cells engage in complex reciprocal interactions, exchanging signals that can shift the balance toward either a diseased or a healthy state. This bidirectional communication blurs the boundaries between mutant and wildtype phenotypes, resulting in intermediate cellular states. Importantly, this principle extends beyond RTT as phenotypic convergence has also been observed in mosaic Huntington’s disease telencephalic organoids [109] and mosaic *Cldn5* knockout brain endothelial cells [110]. Similar intercellular dynamics may also occur in other mosaic disorders, including X-linked diseases (e.g., DMD and FXS) and conditions characterized by somatic mosaicism (e.g., cancer [111], Alzheimer’s disease [112], and autism spectrum disorder [113]).

The intercellular crosstalk in a mosaic environment has profound implications for disease modeling and therapeutic development. Particularly, reductionist models that focus exclusively on homogeneous cell populations fail to capture the emergent properties of the mosaic brain, potentially leading to drugs that target cellular states that do not exist in vivo. Advances in in vitro systems, such as brain organoids, now make it possible to incorporate both genetic [109, 114, 115] and cellular diversity [116] within a single model. These systems also allow longitudinal tracking of developmental processes [117–119], providing an opportunity to assess, for instance, the dynamics of secondary XCI skewing. In parallel, single-cell and spatial transcriptomics are enabling detailed characterization of complex heterogeneous tissues. So, the tools to study mosaicism in RTT and related disorders are now within reach. The next challenge lies in applying them to uncover how cellular interactions shape the mosaic brain. Embracing mosaicism as a central feature, rather than a confounding factor, will be essential for advancing our understanding of mosaic conditions. It also carries translational implications, paving the way for therapeutic strategies tailored to the heterogeneous cellular landscape in mosaic conditions.

## Data Availability

No datasets were generated or analysed during the current study.

## Abbreviations

DMD: Duchenne muscular dystrophy
ESC: embryonic stem cell
EV: extracellular vesicle
FXS: fragile X syndrome
hESC: human embryonic stem cell
hiPSC: human induced pluripotent stem cell
lncRNA: long non-coding RNA
NPC: neural progenitor cells
RTT: Rett syndrome
XCI: X chromosome inactivation
